# Congenital Chylothorax

**DOI:** 10.1100/tsw.2009.62

**Published:** 2009-06-12

**Authors:** Saad Lahmiti, Jamila Elhoudzi, Salwa Baki, Abdelmounaim Aboussad

**Affiliations:** ^1^ Neonatal Intensive Care Department, Mohammed VI University Hospital, Marrakech, Morocco; ^2^ Research Team for Childhood, Health and Development, MarrakechSchool of Medicine, Cadi Ayyad University, Marrakech, Morocco

**Keywords:** chyle, chylothorax, newborn, enteral nutrition

## Abstract

Although pleural effusion is a rare cause of respiratory distress in newborns, being familiar with this disease is very important because of the generally favorable prognosis when the diagnosis is done early and therapy is prompt. We report a case of a full-term baby diagnosed with respiratory distress after 1 week of life. An X-ray of his chest showed a left pleural effusion. Moreover, a thoracentesis combined with a biochemical study of the pleural fluid confirmed the diagnosis of chylothorax. In our case, the conservative therapy was successful. The baby was followed up for a period of 6 months, with no evidence of recurrence. We have concluded, therefore, that conservative management should be the first line of treatment in chylothorax cases. If it does not work, a surgical approach might be considered.

## INTRODUCTION

Congenital chylothorax is the accumulation of lymphatic fluid within the pleural space. It is an uncommon cause of respiratory distress in the neonate. Initially, it is diagnosed during prenatal ultrasound visits or postnatal X-rays, and is confirmed by evaluation of the pleural fluid. The etiology is not well understood and reaccumulation of fluid can occur. Thoracentesis and chest tube placement may be required to maintain a normal respiratory status. A conservative treatment is mostly based on a diet therapy. It may last for up to 5 weeks and should be attempted before any surgical intervention.

## CASE PRESENTATION

A male baby was delivered vaginally at 38 weeks of gestation with a birth weight of 3700 g to a 28-year-old mother. During his first week of life, the baby showed no respiratory distress. After that, he started to have feeding difficulties and had cyanotic episodes during breast-feeding. He was seen by a doctor and then referred to our ward. Physical examination on admission revealed respiratory distress quoted at 4/10 on the Silverman score and decreased breath sounds on the left side. Saturation of oxygen was 94% in 1 l/min of oxygen. X-ray of the chest (Fig. 1) revealed left-sided pleural effusion. Therapeutic thoracentesis removed 85 ml of an orange fluid (Fig. 2) with the following characteristics: protein 4 g/100 ml, triglycerides 52 mg/100 ml, and 92% lymphocytes. Gram stain showed no bacteria and the culture was sterile after 7 days.

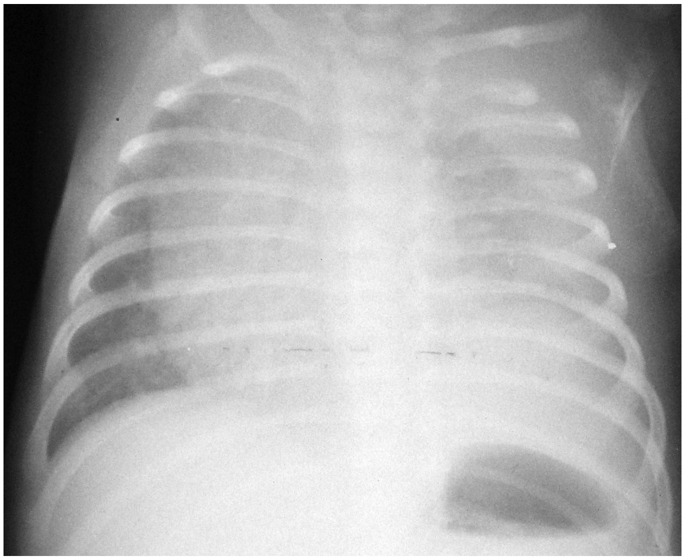

**FIGURE 1.** Chest X-ray showing a left pleural effusion.

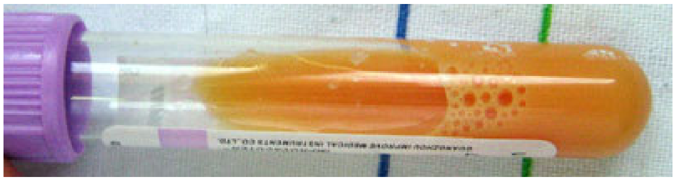

**FIGURE 2.** Orange pleural fluid.

Chylothorax was diagnosed. The respiratory distress significantly decreased just after the thoracentesis was performed and it totally disappeared after 24 h. Artificial ventilation was not needed in this case. He was discharged home after 3 weeks with a formula containing a medium-chain triglyceride, without any respiratory symptoms. One month later, an X-ray of the chest (Fig. 3) showed no further accumulation of fluid and within a period of 6-months, there were no signs of recurrence at follow-up visits.

## DISCUSSION

Chylothorax is the accumulation of lymphatic fluid within the pleural space. It has been recognized as a clinical entity since the first report by Asellius in 1627–1628[1]. Pisek, and Stewart and Linner, were the first to recognize spontaneous chylothorax in newborns in 1917 and 1926, respectively[2].

This condition is usually associated with birth trauma. It is believed that birth trauma may result in a tear of the major duct leading to persistent pleural effusion[3]. In 50% of cases, symptoms of congenital chylothorax are present at birth, but they can also, as in our case, develop during the first week of life. In some cases, a congenital chylothorax is associated with Turner syndrome, Noonan syndrome, hydrops fetalis, or Down syndrome[4]. It is also associated with congenital goiter, lung tumors, congenital lymphangiectasis, pulmonary sequestration, congenital cytomegalovirus and adenoviral infections, right diaphragmatic hernia, and group B streptococcal infections[5]. In our case, no specific etiology was found.

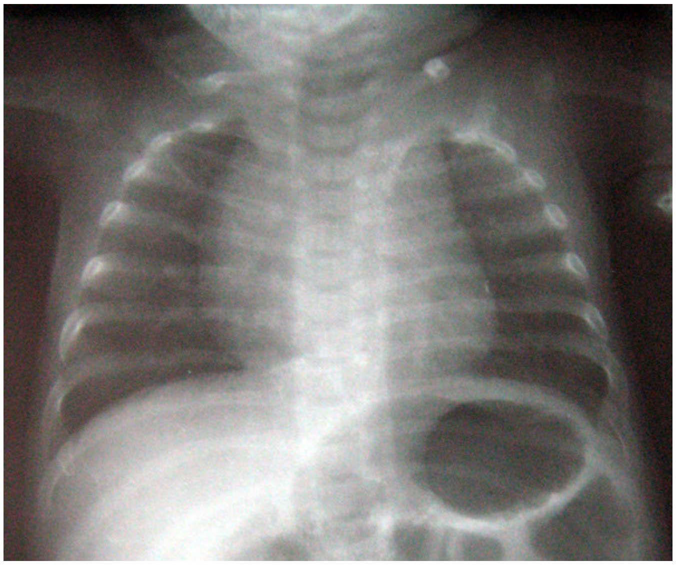

**FIGURE 3.** Chest X-ray at 2 months showing no pleural effusion.

Criteria for the diagnosis of chylothorax are the following: pleural fluid protein concentration >20 g/l, triglyceride concentration >100 mg/dl, number of cells per milliliter >100 with lymphocyte predominance, and sterile culture[6]. A milky appearance of the fluid (indicating presence of chylomicrons) and a positive Sudan III test result confirm the diagnosis in orally fed infants[7]. In patients not receiving enteral nutrition, elevated triglyceride levels are frequently not detected in pleural fluid analysis. The prevalence of lymphocytes in the drainage fluid is of major importance in the diagnosis since this finding is independent of nutrition[8].

Usually, initial therapy for postoperative chylothorax has always been pleural space drainage, use of medium-chain triglyceride oil, fat-free baby formula, or enteric rest with total parenteral nutrition (TPN)[9]. In our patient, a fat-free formula consisting of proteins and starch was given, and led to effusion abating within 3 weeks. If effusion does not abate after 2–3 weeks, TPN should be started together with total enteric rest. During the last several years, octreotide, a somatostatin analogue, has become another option for conservative management of chylothorax[7]. Surgery is usually not considered prior to the initial therapy. In fact, most authors recommend an extended period of conservative treatment and do not proceed to a surgical approach until after 4 weeks of pleural effusion. The surgical procedure includes pleurodesis, the ligation of the main duct with adjacent leaking lymphatics[9].

## CONCLUSION

Chylothorax is a rare cause of respiratory distress in newborns. Its diagnosis is based on clinical, radiological, and pleural fluid biochemical findings. Conservative therapy should be tried at first, but if it does not succeed, a surgical procedure might be considered.
